# Supplementary material for: p53 wild-type colorectal cancer cells that express a fetal gene signature are associated with metastasis and poor prognosis
Source: Nat Commun. 2022 May 23;13:2866. doi: 10.1038/s41467-022-30382-9 (PMC9126967; doi:10.1038/s41467-022-30382-9)
Supplement: Supplementary file 1 — Supplementary Information File [file 41467_2022_30382_MOESM1_ESM.pdf]

Supplementary materials for

p53 wild type colorectal cancer cells that express a fetal gene signature are associated with metastasis and poor prognosis.

Laura Solé, Teresa Lobo-Jarne, Daniel Alvarez-Villanueva, Josune Alonso-Marañón, Yolanda Guillén, Marta Guix, Irene Sangrador, Catalina Rozalén, Anna Vert, Antonio Barbachano, Joan Lop, Marta Salido, Beatriz Bellosillo, Raquel García-Romero, Marta Garrido, Jessica González, María Martínez-Iniesta, Erika Lopez-Arribillaga, Ramón Salazar, Clara Montagut, Ferrán Torres, Mar Iglesias, Toni Celià-Terrassa, Alberto Muñoz, Alberto Villanueva, Anna Bigas* and Lluís Espinosa*.

* Correspondence should be addressed to A.B. or L.E. (abigas@imim.es; lespinosa@imim.es)

This PDF file includes:
Supplementary figures 1 to 6 and
Supplementary tables 1 to 6

Reagents, antibodies and software

A table of the source of all reagents, antibodies, kits, cell lines, chemicals and software are included (Table S4).

Supplementary Figures, Tables and Data:

Fig. S1. Low-dose CT treatment induces a quiescent-like state to CRC PDO in the absence of persistent DNA damage and senescence.

Fig. S2. PQL cells retain tumor initiating capacity.

Fig. S3. Low-dose CT induces a robust p53 signaling.

Fig. S4. Acquisition of a quiescent phenotype by CT treatment in patients.

Fig. S5. Acquisition of a restricted fetal ISC signature by CT and p53 dependency.

Fig. S6. Identification of a fetal ISC signature with prognostic value in cancer.

Table S1. Patient-derived organoids used in this study.

Table S2. Human gastrointestinal tumor samples used in this study.

Table S3. Cox proportional hazards analysis of the feISC signature..

Table S4. Materials table.

Table S5. List of oligonucleotides for RT-qPCR and ChIP-qPCR and sgRNA for CIRSPR/Cas9 knockout used in this study.

Table S6. Tissue microarray (TMA) samples data.

Data 1. Differentially expressed genes between IC20 or IC30 and untreated PDOs.

Data 2. Expression correlation matrix from CT induced feISC genes in the Marisa dataset.

Supplementary References

1. Kim D, Pertea G, Trapnell C, et al. TopHat2: Accurate alignment of transcriptomes in the presence of insertions, deletions and gene fusions. Genome Biol 2013.
2. Anders S, Pyl PT, Huber W. HTSeq-A Python framework to work with high-throughput sequencing data. Bioinformatics 2015.
3. Mulero M, Ferres-Marco D, Islam A, et al. Chromatin-bound IκBα regulates a subset of polycomb target genes in differentiation and cancer. Cancer Cell 2013;24.
4. Krueger B, Friedrich T, Förster F, et al. Different evolutionary modifications as a guide to rewire two-component systems. Bioinform Biol Insights 2012.
5. Langmead B, Salzberg SL. Fast gapped-read alignment with Bowtie 2. Nat Methods 2012.
6. Zhang Y, Liu T, Meyer CA, et al. Model-based analysis of ChIP-Seq (MACS). Genome Biol 2008.
7. Yu G, Wang LG, He QY. ChIP seeker: An R/Bioconductor package for ChIP peak annotation, comparison and visualization. Bioinformatics 2015;31:2382–2383.
8. Sato T, Stange DE, Ferrante M, et al. Long-term expansion of epithelial organoids from human colon, adenoma, adenocarcinoma, and Barrett’s epithelium. Gastroenterology 2011.

Supplementary Figures and Legends

Figure S1. Low-dose CT treatment induces a quiescent-like state to CRC PDO in the absence of persistent DNA damage and senescence

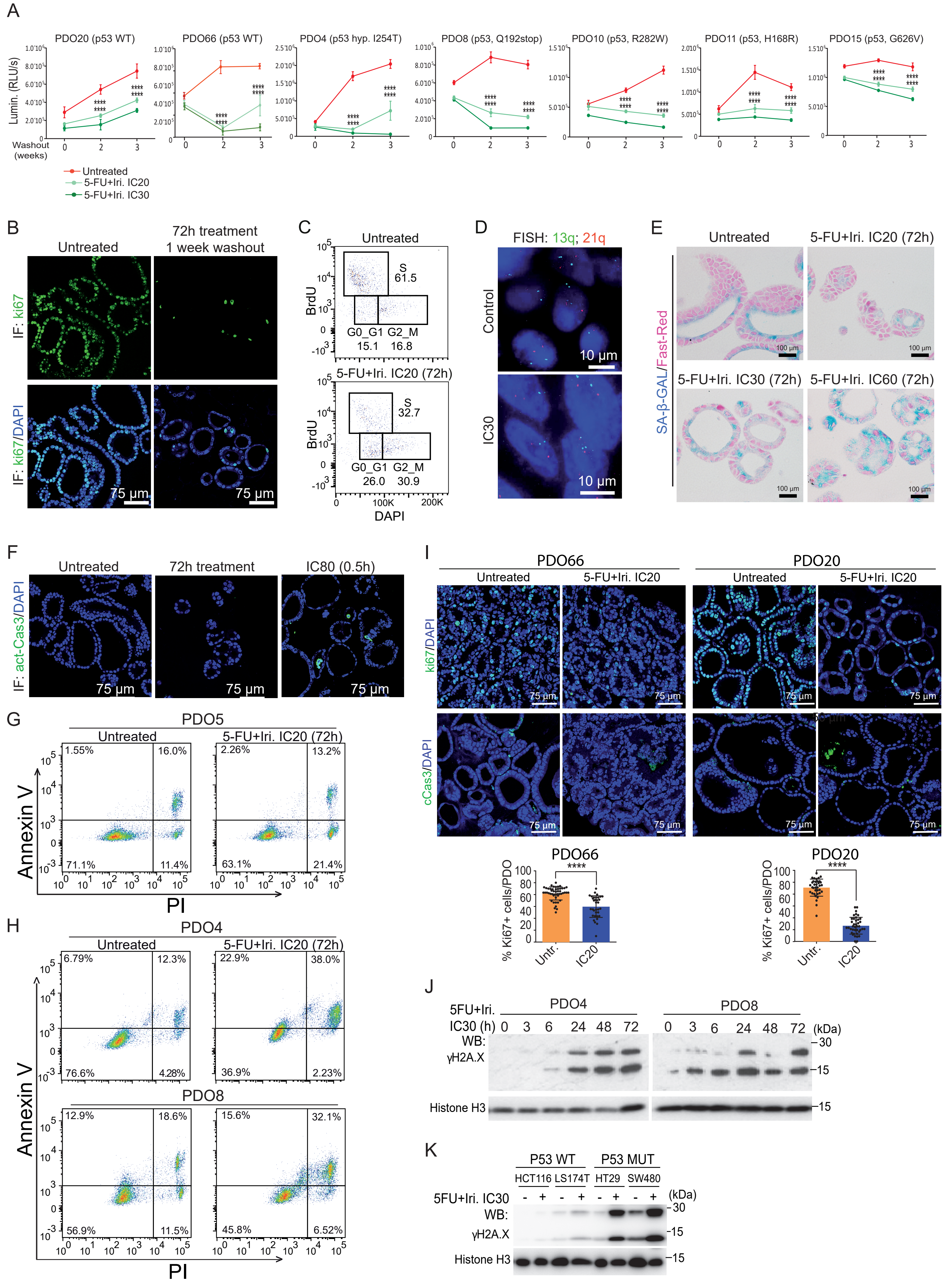

Figure S1. Related to Figure 1. Low-dose CT treatment induces a quiescent-like state to CRC PDO in the absence of persistent DNA damage and senescence.

(A) Quantification of cell viability of the different PDOs untreated or pretreated with 5-FU+Iri. for 72 hours and then maintained in fresh medium for 1-2 weeks after washout (n = 3 replicates, from 1 out of 3 biologically independent experiments). **(B)** Representative images of the proliferation marker ki67 staining by IF in PDO5 tumoroid treated with 5-FU+Iri. at IC_{20} for 72 hours and after being maintained in fresh medium for 1 week (from 1 out of 3 biologically independent experiments). **(C)** Flow cytometry analysis showing BrdU incorporation of PDO5 after 72 hours of 5-FU+Iri. treatment, compared with the untreated cells. Boxes represent cells in G_0/G_1, S and G_2/M cell cycle, respectively. **(D)** Representative images of FISH analysis from untreated and IC_{30}-treated PDO5 using probes for 13q (green) and 21q (red) (from 1 out of 2 biologically independent experiments). **(E)** Analysis of SA-β-Gal activity in PDO5 cells treated with 5-FU+Iri. as indicated for 72 hours. Representative images were obtained with Olympus BX61 (from 1 out of 2 biologically independent experiments). **(F)** Representative IF images of cleaved-caspase 3 staining in PDO5 treated with 5-FU+Iri. at IC_{20} at the indicated time points and with IC_{80} as a positive control (from 1 out of 3 biologically independent experiments). **(G and H)** Cytometry analysis of Annexin V binding in PDO5 **(G)**, PDO4 and PDO8 **(H)** untreated or treated as indicated. **(I)** Representative IF images of ki67 and cleaved-caspase 3 staining in PDO66 and PDO20 treated with 5-FU+Iri. at IC_{20} at the indicated time points (n = 35 spheres examined over 3 biologically independent experiments). **(J and K)** WB analysis of PDOs **(J)** and CRC cell lines **(K)** treated as indicated (from 1 out of 3 biologically independent experiments).

For all applicable figures, data are mean ± SD. Significance (p) was calculated in I with two-sided Student's t-test and in A with two-way ANOVA test. ****$p < 0.0001$. SA-β-Gal, SA-β-Galactosidase; 5-FU, 5-fluorouracil; Iri, irinotecan; CT, control; IC_{20} and IC_{30}, 5-FU+Iri. treatment that results in 20% and 30% cell death, respectively, compared with untreated. Source data are provided as a Source Data file.

Figure S2. PQL cells retain tumor initiating capacity

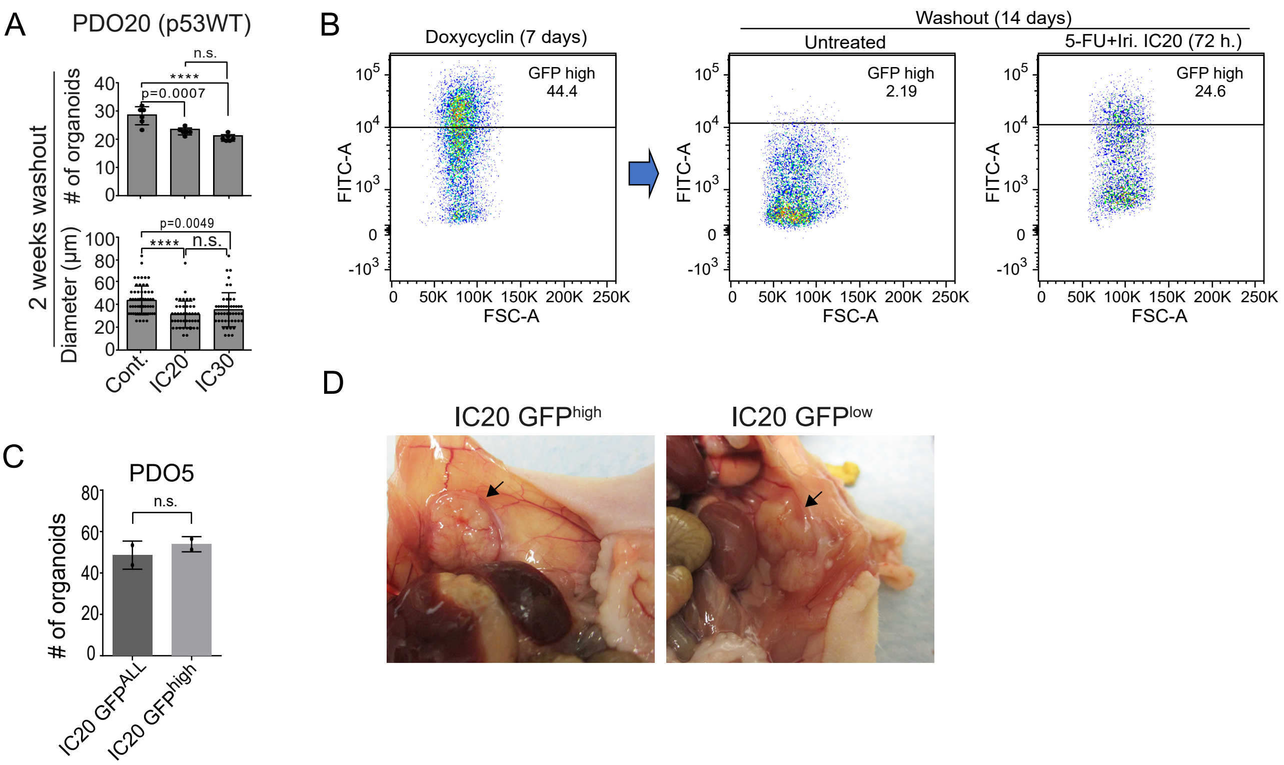

Figure S2. Related to Figure 2. PQL cells retain tumor initiating capacity.

(A) Number of PDOs (upper panel) and diameter (lower panel) of *TP53* WT PDO20 treated with 5-FU+Iri. as indicated, and left for 2 weeks with fresh medium. 300 cells/well were seeded (n = 6 wells examined for TICs and n = more than 45 spheres examined whenever possible for diameters, from 3 biologically independent experiments). **(B)** Analysis of GFP distribution by flow cytometry of PDO5-hG. Cells were treated for 6 days with doxycycline to induce GFP-H2B expression and then left untreated or treated with 5-FU+Iri. IC_{30} for 72 hours and maintained in fresh medium for 2 additional weeks. Quiescent cells that retained high or low GFP levels were purified by cell sorting. **(C)** Number of PDOs generated from seeding 300 $GFP^{high+low}$ and GFP^{high} sorted PDO5-hG cells after 2 weeks with fresh medium (n = 6 wells examined for TICs and n = more than 50 spheres examined whenever possible for diameters, from 3 biologically independent experiments). **(D)** Representative pictures of intraperitoneal implants (arrows) detected in nude mice orthotopically implanted with IC_{20}-pretreated PDO5-hG GFP^{high} or GFP^{low} sorted cells.

For all applicable figure panels, data are mean ± SD. Significance (p) was calculated in A with one-way ANOVA test and in C with two-sided Student's t-test test. ****$p < 0.0001$; n.s., no significant. 5-FU, 5-fluorouracil; Iri, irinotecan; IC_{20}, 5-FU+Iri. treatment that results in 20% cell death, compared with untreated cell growth. Source data are provided as a Source Data file.

Figure S3. Low-dose CT induces a robust p53 signaling

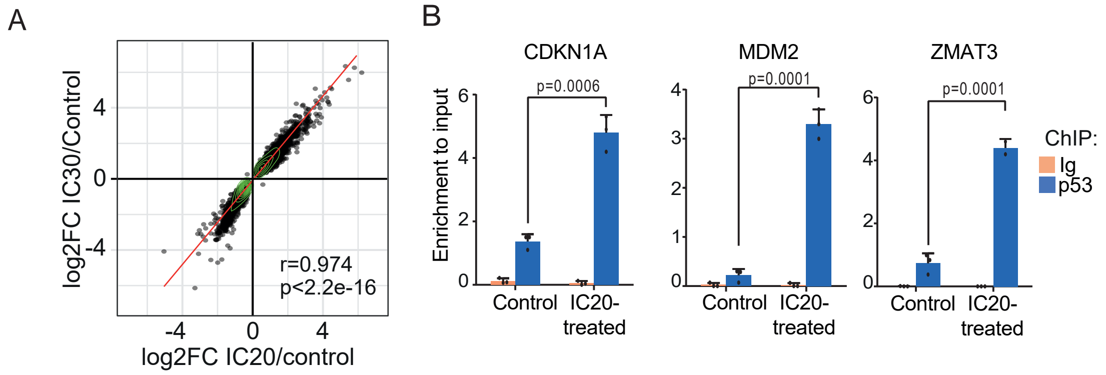

Figure S3. Related to Figure 3. Low-dose CT induces a robust p53 signaling.

(A) Linear association of the genes differentially expressed in treated PDO5 compared with the control. Dots represent the log2 fold change values of genes for IC20 compared with control (x-axis) and IC30 compared with control (y-axis). The Pearson correlation and p value are shown. **(B)** ChIP-qPCR analysis of p53 binding in untreated and IC20-treated PDO5 in a subset of putative p53 target genes expressed as relative enrichment normalized to the input (a validation of the ChIP-seq).

For all applicable figure panels, data are mean ± SD. Significance (p) was calculated with two-sided Student's t-test. CT, IC20 and IC30, 5-FU+Iri. treatment that results in 20% and 30% cell death, respectively, compared with untreated cell growth. Source data are provided as a Source Data file.

Figure S4. Acquisition of quiescent phenotype by CT treatment in patients.

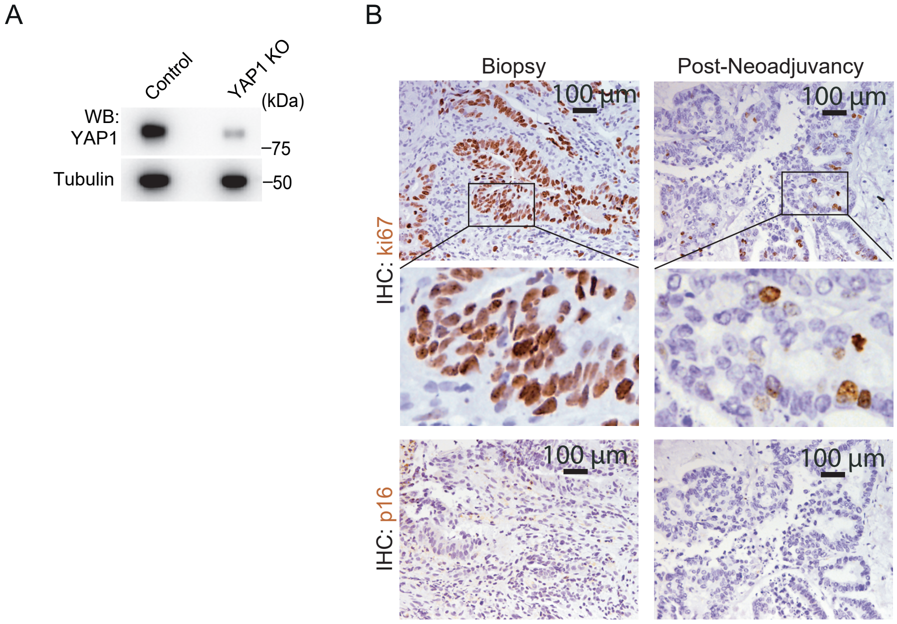

Figure S4. Related to Figure 4. Acquisition of quiescent phenotype by CT treatment in patient samples.

(A) WB analysis of PDO5 control and *YAP1* KO (from 1 out of 3 biologically independent experiments). **(B)** IHC analysis of ki67 and p16 in representative type 2 colorectal tumor samples (Supplementary Table S2 #17) from the same patient at diagnosis (biopsy) and after neoadjuvant therapy at the time of surgery (post-neoadjuvancy) (from 1 out of 6 biologically independent samples).

Figure S5. Restricted 28up + 8 down feISC signature and p53 dependency

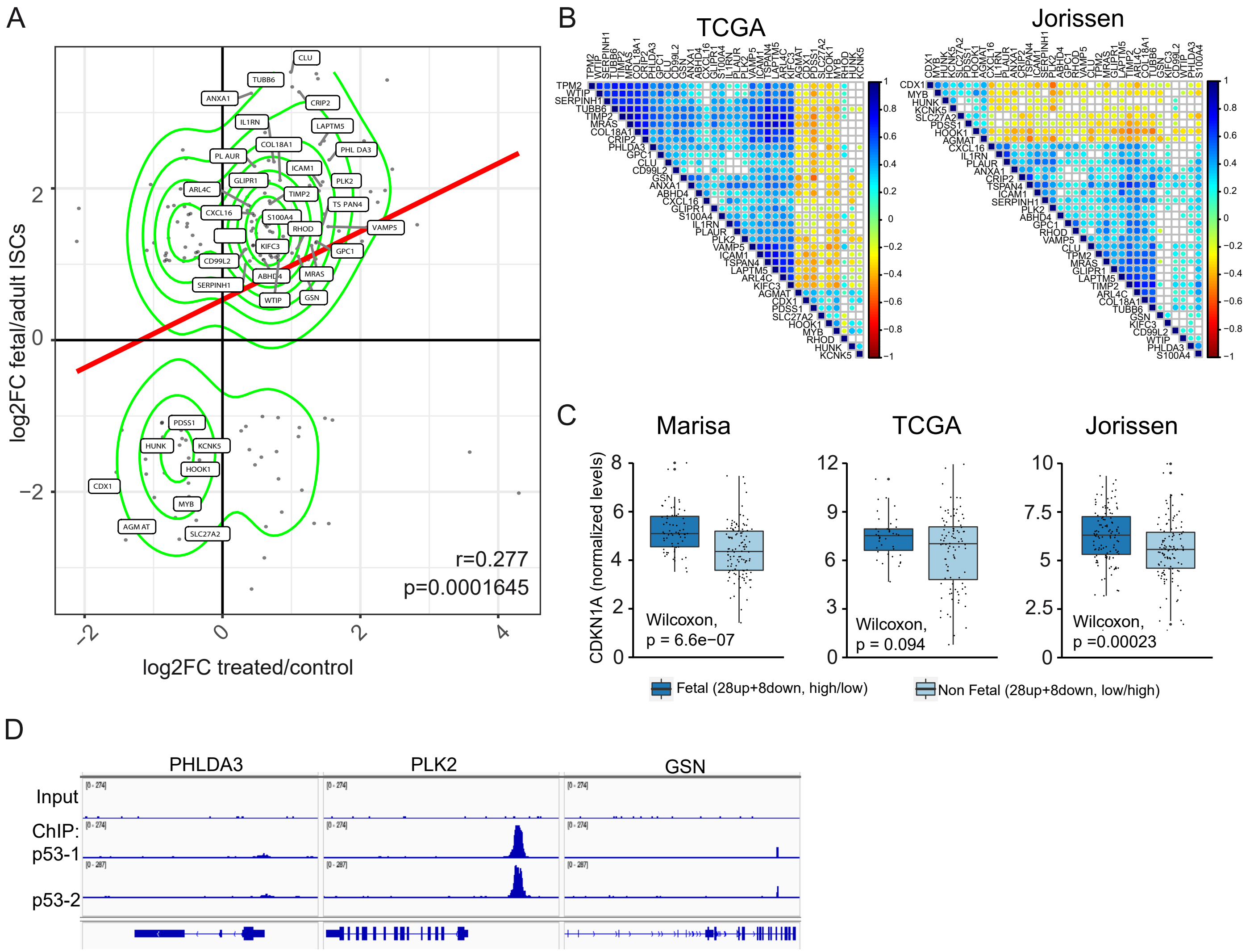

Figure S5. Related to Figure 5. Acquisition of a restricted feISC signature by CT and p53 dependency.

(A) Scatter plot and linear regression line of the genes differentially expressed between CT treated and control PDOs and fetal compared with adult ISC. Dots represent the log2 fold change values of genes for treated versus control (x-axis) and fetal versus adult ISC (y-axis). The Pearson correlation and p-value are shown. Genes included in the 28up+8down-feISC signature are indicated. **(B)** Expression correlation matrix from the 28up+8down-feISC gene signature in TCGA (n=329) and Jorissen (n=226) databases. Size of circles and color intensity are proportional to Pearson correlation coefficient for each gene pair. **(C)** Box plots of *CDKN1A* levels in tumors classified as 28up+8down-feISC (fetal type or non-fetal type) from the Marisa (fetal n=66, non-fetal n=114), Jorissen (fetal n114, non-fetal n=112),and TCGA (fetal n=39, non-fetal n=96), datasets. Boxes represent the central 50% of the data (from the lower 25th percentile to the upper 75th percentile), lines inside boxes represent the median (50th percentile), and whiskers are extended to the most extreme data point. Statistical p value from Wilcoxon two-sided test is shown. **(D**) Representation of p53 distribution in the indicated genomic regions of 28up feISC genes obtained from ChIP-sequencing analysis in IC_{20}-treated PDO5 (n=2).

Figure S6. Identification of a fetal ISC signature with progonostic value in *TP53* WT CRC

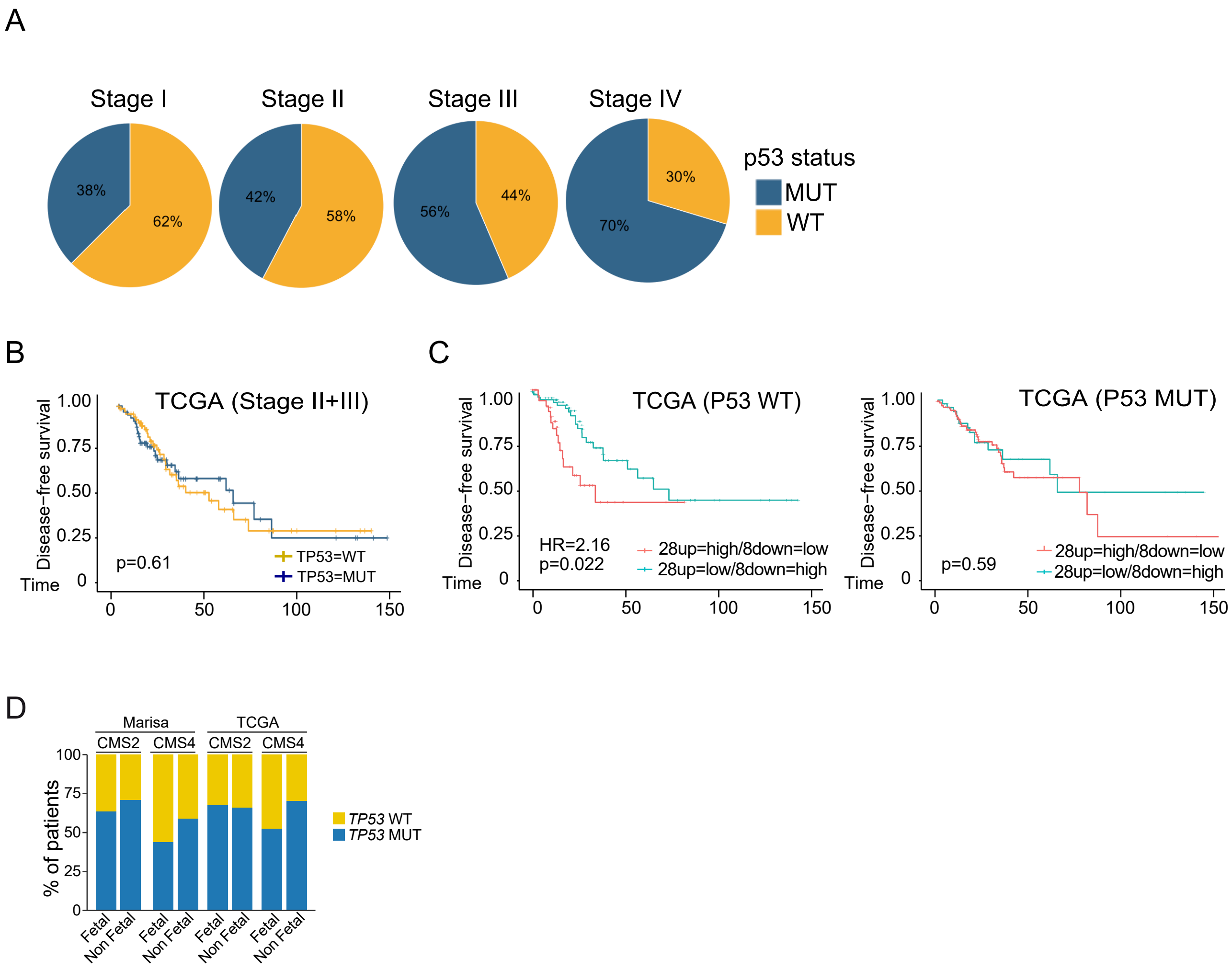

Figure S6. Related to Figure 6. Identification of a fetal ISC signature with prognostic value in cancer.

(A) Distribution of tumors from the TCGA colorectal cancer dataset carrying WT and mutated *TP53* inside the different tumor stages. **(B)** Kaplan-Meier representation of disease-free survival probability over time of patients classified according to their *TP53* status (*TP53* WT n=144 and *TP53* MUT n=177) in the TCGA dataset. **(C)** Kaplan-Meier representation of disease-free survival probability over time of patients, from the TCGA dataset, classified according to their cluster analysis of the 28up+8down-feISC signature (data not shown) for patient groups from *TP53* WT (*n=144*) and *TP53* mutant (*n*=177). (D) Percent of tumors with *TP53* WT or *TP53* mutant alleles in the CMS2 and CMS4 subgroups from Marisa and TCGA datasets, previously classified according to the 28up+8down-feISC signature. Fetal: 28up=high/8down=low; non-Fetal: 28up=low/8down=high.

For statistical analysis of the Kaplan-Meier estimates we used Cox proportional hazards models analysis and logrank two-sided p value (See Supplementary Table S3). HR, hazard ratio.

Supplementary Table S1. Patient-derived organoids and CRC cells lines used in this study. The mutations and the corresponding chemotherapy concentrations that reduce a 20 and 30% of the cell growth (IC_{20} and IC_{30}, respectively) are indicated for each PDO and cell line.

Cell line	Mutations	IC_{20} (µg/mL)	IC_{30} (µg/mL)	Source
PDO4	TP53 I254T (100%)<br>EGFR S464L (97.21%)	5-FU 1.25<br>Iri. 0.50	5-FU 2.00<br>Iri. 0.80	Primary tumor
PDO5	KRAS G12D (66.43%)	5-FU 0.14<br>Iri.] 0.06	5-FU 0.25<br>Iri. 0.10	Primary tumor
PDO5 (p53 KO)	KRAS G12D (66.43%)	5-FU 0.25<br>Iri. 0.10	n.d.	Derived from PDO5
PDO5 (YAP1 KO)	KRAS G12D (66.43%)	n.d.	5-FU 0.14<br>Iri. 0.06	Derived from PDO5
PDO8	TP53 Q192stop (98.46%)<br>KRAS G13C (67.27%)	5-FU 0.78<br>Iri. 0.31	5-FU 1.56<br>Iri. 0.63	Primary tumor
PDO10	TP53 R282W (99.82)<br>FGFR2 C809W (62.72%)<br>KRAS A146V (80.25%)	5-FU 6.25<br>Iri. 2.50	5-FU 12.50<br>Iri. 5.00	Primary tumor
PDO11	TP53 H168R (46.54%)<br>FGFR2 N194stop (45.87%)<br>KRAS G12D (49.58%)<br>ERBB2 A87T (4.76%)<br>PIK3CA S874N (41.93%)<br>PDGFRA R293H (5.97%)<br>EGFR K960R (46.3%)<br>BRAF E71D (42.08%)	5-FU 0.78<br>Iri. 0.31	5-FU 1.56<br>Iri. 0.63	Liver metastasis. CT and Cetuximab resistant
PDO15	TP53 G262V (98.82%)	5-FU 1.56<br>Iri. 0.63	5-FU 3.13<br>Iri. 1.25	Ovarian metastasis. CT and Cetuximab resistant
PDO66	NRAS (G12S) (99.05%)<br>APC (S1110stop) (99.80)	5-FU 0.63<br>Iri. 0.30	5-FU 2.50<br>Iri. 1.00	Primary tumor
PDO20	APC Q1123X (60.38%)<br>KRAS G12V (26.42%)<br>PI3KCA H1047L (49.94%)	5-FU 0.25<br>Iri. 0.10	5-FU 0.50<br>Iri. 0.20	Primary tumor
HCT116	TP53 WT	n.d.	5-FU 0.2<br>Iri. 0.08	Cell line
Ls174T	TP53 WT	n.d.	5-FU 0.3<br>Iri. 0.13	Cell line
LoVo	TP53 WT	n.d.	5-FU 0.06<br>Iri. 0.02	Cell line
DLD1	TP53 S241F; -	n.d.	5-FU 0.12<br>Iri. 0.05	Cell line
SW480	TP53 R273H, P309S	n.d.	5-FU 3.33<br>Iri. 1.33	Cell line
HT29-M6	TP53 R273H	n.d.	5-FU 3.33<br>Iri. 1.33	Cell line

Supplementary Table S2. Human gastrointestinal tumor samples used in this study. Paired samples at diagnosis (biopsy) and after neoadjuvant therapy at the time of surgery (postQ). The corresponding clinical data, ki67 subtype classification (1. No changes in ki67 levels; 2. Decrease in ki67 levels; 3. Decrease in ki67 levels displaying giant nuclei) and presence of nuclear YAP1 is indicated.

Patient nº	Tumor Localization	Clinical TNM	Treatment	ki67% postQ	ki67% biopsy	ki67 subtype	nuclear YAP1% postQ	nuclear YAP1% biopsy	OS (mo)	DFS (mo)
**1**	Gastric	T4N1M1	Chemotherapy	20	40	2	30	0	7,13	5,40
**2**	Colorectal	T3N1M1	Chemotherapy + Targeted therapy	1	55	3	N/A	N/A	115,23	115,23
**3**	Gastric	T3N1	Chemotherapy	10	70	2	90	5	60,90	60,90
**4**	Colorectal	T3N1M1	Radiotherapy	5	70	2	N/A	N/A	17,33	9,50
**5**	Gastric	T3N1M0	Chemotherapy	5	60	2	80	5	26,73	12,23
**6**	Colorectal	T3N0	Chemotherapy	30	70	2	50	2	53,10	53,10
**7**	Gastric	T3N1M1	Chemotherapy	70	90	2	90	80	41,10	23,07
**8**	Gastric	T3N1M1	Chemotherapy + Targeted therapy	10	5	1	70	0	89,83	40,23
**9**	Colorectal	T3N1	Chemotherapy	15	75	2	N/A	N/A	94,23	94,23
**10**	Gastric	T3N0M0	Chemotherapy	85	95	1	5	10	91,10	91,10
**11**	Gastric	T2-3N1M0	Chemotherapy	90	90	1	N/A	N/A	93,37	93,37
**12**	Gastric	T3N1M0	Chemotherapy	80	90	1	N/A	N/A	97,70	97,70
**13**	Gastric	T3N0M0	Chemotherapy	20	70	2	0	0	25,23	9,67
**14**	Colorectal	T4N1	Chemotherapy	80	60	1	95	20	38,17	12,43
**15**	Gastric	T3N1M1	Chemotherapy + Targeted therapy	70	85	1	50	15	93,37	93,37
**16**	Colorectal	T3N1	Chemotherapy + Targeted therapy	10	80	2	90	0	42,67	21,67
**17**	Colorectal	T4N1M0	Chemotherapy	20	95	2	60	20	39,90	39,90
**18**	Colorectal	T3N0M0	Chemotherapy	40	90	2	N/A	N/A	81,53	81,53
**19**	Colorectal	T2N1M0	Chemotherapy	5	25	3	90	3	83,13	83,13
**20**	Colorectal	T4N1M0	Chemotherapy	20	60	2	70	60	94,03	94,03
**21**	Colorectal	T3N1M0	Chemotherapy	25	80	2	N/A	30	91,90	91,90
**22**	Colorectal	T3N1M0	Chemotherapy	1	95	2	N/A	N/A	88,30	88,30
**23**	Colorectal	T3N2bM0	Chemotherapy	1	90	2	30	30	76,53	76,53
**24**	Gastric	T3N1M0	Chemotherapy	20	20	1	90	0	29,87	12,77
**25**	Colorectal	T3N1M0	Chemotherapy	N/A	N/A	N/A	N/A	N/A	79,77	N/A
**26**	Colorectal	T3N0M0	Chemotherapy	N/A	N/A	N/A	N/A	N/A	53,63	N/A
**27**	Colorectal	T3N0M0	Chemotherapy	1	55	2	5	10	8,53	8,53
**28**	Colorectal	T3N1M0	Chemotherapy + Targeted therapy	40	65	2	30	1	121,23	27,47
**29**	Colorectal	T3N1M0	Chemotherapy	N/A	N/A	N/A	N/A	N/A	33,90	20,83
**30**	Colorectal	T3N0	Chemotherapy	20	10	1	70	3	60,30	60,30

**31**	Gastric	T3N0	Chemotherapy	20	30	1	100	0	12,17	9,10
**32**	Gastric	T3N1	Chemotherapy	30	40	1	N/A	N/A	16,53	8,40
**33**	Gastric	T3N0M0	Chemotherapy	30	70	2	80	5	77,93	77,93
**34**	Colorectal	T3N1M1	Chemotherapy + Targeted therapy	5	80	2	90	0	N/A	N/A
**35**	Colorectal	T3N1M0	Radiotherapy	1	60	2	100	15	69,50	69,50
**36**	Gastric	T3N1M0	Chemotherapy	25	N/A	N/A	N/A	N/A	39,37	39,37
**37**	Gastric	T3N1M0	Chemotherapy	20	55	2	2	2	65,10	65,10
**38**	Gastric	T3N1M0	Chemotherapy	10	20	1	60	20	39,23	22,73
**39**	Pancreas	T4N0M0	Chemotherapy	0	N/A	N/A	N/A	N/A	45,63	15,63
**40**	Colorectal	T3N1M1	Chemotherapy	25	30	1	90	10	18,23	6,00
**41**	Colorectal	T3N0M0	Radiotherapy	25	40	1	30	0	41,77	41,77
**42**	Colorectal	T3N0M1	Chemotherapy + Targeted therapy	40	90	2	90	0	22,50	10,13
**43**	Colorectal	T4N1M0	Chemotherapy	0	65	2	0	5	45,60	11,90
**44**	Colorectal	T3N1	Chemotherapy	5	15	1	0	5	8,70	8,70
**45**	Colorectal	T3N0M0	Chemotherapy	1	70	2	N/A	N/A	62,73	25,00
**46**	Colorectal	T3N1M1	Chemotherapy + Targeted therapy	5	50	2	80	0	33,47	18,43
**47**	Colorectal	T3N2M0	Chemotherapy	60	95	2	0	2	59,63	26,40
**48**	Colorectal	T3N1M0	Chemotherapy	20	60	2	30	0	56,20	56,20
**49**	Gastric	T4N0M0	Chemotherapy	50	60	1	50	50	42,70	42,70
**50**	Colorectal	T3N0M0	Chemotherapy	10	70	2	5	0	47,60	47,60
**51**	Gastric	T2N1M0	Chemotherapy	15	75	2	2	0	38,73	16,70
**52**	Colorectal	T3N0M0	Chemotherapy	5	25	2	30	0	63,37	63,37
**53**	Colorectal	T3N1M0	Chemotherapy	20	95	2	30	0	9,00	9,00
**54**	Gastric	T3N3M0	Chemotherapy	15	60	2	30	0	11,93	11,03
**55**	Colorectal	T4N1M0	Radiotherapy	20	30	1	15	0	34,73	34,73
**56**	Colorectal	T2N1M0	Chemotherapy	N/A	N/A	N/A	N/A	N/A	53,27	N/A
**57**	Colorectal	T3N1M0	Chemotherapy	45	85	2	50	5	54,47	54,47
**58**	Colorectal	T3N0M0	Chemotherapy	70	90	2	90	20	50,67	50,67
**59**	Colorectal	T3N0M0	Chemotherapy	45	80	2	70	25	12,23	5,00
**60**	Colorectal	T4N2M0	Chemotherapy	75	95	2	40	0	22,47	4,27
**61**	Colorectal	T3Nx (sigma)+ T2N0 (rectum) M1	Radiotherapy	65	65	1	N/A	N/A	36,47	3,27
**62**	Colorectal	T3N0 (rectum) + T4Nx (bladder) M1	Radiotherapy	80	95	1	80	5	5,37	3,53

Table S3 Cox proportional hazards analysis of the feISC signature. Association of the signature with recurrence-disease free survival. Related to Figure 5 and Figure S5.

**GSE39582** (Marisa et al)	**28up=high 8down=low**	**p value (logrank test)**	**HR recurrence**	**lower.95 (HR)**	**upper.95 (HR)**
All stages, *n=566*	*n= 66*	8.00E-04	2.33	1.40	3.9
Stage II, *n=149*	*n= 23*	4.10E-02	2.27	1.01	5.1
Stage II-III, *n=468*	*n= 100*	3.30E-03	1.71	1.19	2.5
Stage IV, *n=60*	*n= 25*	2.37E-01	1.48	0.77	2.8
p53WT, *n=85*	*n= 27*	4.67E-02	2.16	1.01	4.6
p53MUT, *n=20*	*n= 6*	9.60E-01	1.04	0.27	4.0
CMS4, *n=91*	*n= 23*	7.60E-02	1.79	0.92	3.46

**TCGA-COAD+READ** (TCGA Portal)	**28up=high 8down=low**	**p value (logrank test)**	**HR recurrence**	**lower.95 (HR)**	**upper.95 (HR)**
All stages, *n=329*	*n= 39*	2.40E-02	2.20	1.09	4.43
P53WT, *n=144*	*n= 47*	2.10E-02	2.16	1.1	4.20
P53MUT, *n=177*	*n= 128*	5.90E-02	1.18	0.63	2.21

**GSE14333** (Jorissen et al)	**28up=high 8down=low**	**p value (logrank test)**	**HR recurrence**	**lower.95 (HR)**	**upper.95 (HR)**
All stages, *n=226*	*n= 114*	1.00E-04	3.28	1.74	6.18

Table S4. Materials table

REAGENT or	SOURCE	IDENTIFIER
**Antibodies**		
Mouse monoclonal anti-γH2AX (pS139) (clone N1-431 (RUO))	BD Biosciences	Cat#564719; RRID:AB_2738913 – (dilution 1/1000)
Mouse monoclonal anti-Ki67 (clone MM1)	Leica Biosystems	Cat#NCL-Ki67-MM1; RRID:AB_442101 – (dilution 1/1000)
Rabbit polyclonal anti-Cleaved Caspase-3 (Asp175)	Cell Signaling	Cat#9661; RRID:AB_2341188 – (dilution 1/1000)
Mouse monoclonal anti-p53 (clone DO-1)	Abcam	Cat#ab1101; RRID:AB_297667 – (dilution 1/1000)
Rabbit monoclonal anti-p21 (clone EPR362)	Abcam	Cat#ab109520; RRID:AB_10860537 – (dilution 1/1000)
Rabbit monoclonal anti-CKN2A/p16INK4a (clone EPR1473)	Abcam	Cat#ab108349; RRID:AB_10858268 – (dilution 1/1000)
Goat polyclonal anti-EphB2	RD Systems	Cat#AF467; RRID:AB_355375 – (dilution 1/1000)
Rabbit polyclonal anti-CD99L2	Abcam	Cat#ab224164 – (dilution 1/1000)
Mouse monoclonal anti-TIMP2 (clone 3A4)	Abcam	Cat#ab1828; RRID:AB_2256129 – (dilution 1/1000)
Rabbit polyclonal anti-MRas	Abcam	Cat#ab26303; RRID:AB_470849 – (dilution 1/1000)
Rabbit polyclonal anti-TUBB6	Abcam	Cat#PA5-P8948 – (dilution 1/2000)
Rabbit monoclonal anti-ICAM1 (clone EPR4776)	Abcam	Cat#ab109361; RRID:AB_10958467 – (dilution 1/1000)
Rabbit monoclonal anti-Hsp47 (clone EPR4217)	Abcam	Cat#ab109117; RRID:AB_10888995 – (dilution 1/1000)
Rabbit monoclonal anti-YAP1 (clone EP1674Y)	Abcam	Cat#ab52771; RRID:AB_2219141 – (dilution 1/1000)
Rabbit polyclonal anti-TSPAN4	Thermo Fisher Scientific	Cat#PA5-69344; RRID:AB_2688603 – (dilution 1/1000)
Mouse monoclonal anti-S100A4 (clone CL0240)	Atlas Antibodies	Cat#AMAB90599; RRID:AB_2665603 – (dilution 1/1000)

Rabbit polyclonal anti-Histone H3 antibody-Nuclear Marker and ChIP Grade	Abcam	Cat#ab1791; RRID:AB_302613 – (dilution 1/10000)
Mouse monoclonal anti-alpha-Tubulin (clone B-5-1-2)	Sigma-Aldrich	Cat#T6074; RRID:AB_477582 – (dilution 1/10000)
Goat Anti-Rabbit Immunoglobulins/HRP antibody (2ary)	Agilent	Cat#P0448; RRID:AB_2617138 – (dilution 1/2000)
Rabbit Anti-Mouse Immunoglobulins/HRP antibody (2ary)	Agilent	Cat#P0260; RRID:AB_2636929 – (dilution 1/2000)
Polyclonal Rabbit Anti-Goat Immunoglobulins/HRP antibody (2ary)	Agilent	Cat#P0449; RRID:AB_2617143 – (dilution 1/2000)
**Biological Samples**		
Patient-derived organoids (PDO): PDO4, PDO5, PDO8, PDO10, PDO11, PDO15	Hospital del Mar (Barcelona)	MARBiobanc (https://marbiobanc.imim.es)
Patient-derived organoids (PDO): PDO66	From Alberto Muñoz Lab (Fernández-Barral et al., 2020)	RetBioH (www.redbiobancos.es)
Human gastrointestinal tumors blocks	Hospital del Mar (Barcelona)	MARBiobanc (https://marbiobanc.imim.es)
**Chemicals, Peptides, and Recombinant Proteins**		
Collagenase II from *Clostridium histolyticum*	Sigma-Aldrich	Cat#C6885
Hyaluronidase from bovine testes	Sigma-Aldrich	Cat#H3506
DMEM/F-12 Advanced	GIBCO	Cat#12634028
Primocin	Invitrogen	Cat#ant-pm-1
B-27 Supplement (50X)	GIBCO	Cat#17504044
N-2 supplement (100X)	GIBCO	Cat#17502048

Nicotinamide	Sigma-Aldrich	Cat#N3376
N-Acetyl-L-cysteine	Sigma-Aldrich	Cat#A7250
Recombinant Human Noggin	PeproTech	Cat#120-10C
Recombinant Human R-Spondin-1	PeproTech	Cat#120-38
Y-27632 dihydrochloride (ROCK inhibitor)	Sigma-Aldrich	Cat#Y0503
Prostaglandin E2	Tocris	Cat#2296
SB 202190	Sigma-Aldrich	Cat#S7067
A8301 (ALK inhibitor)	Sigma-Aldrich	Cat#SML0788
hEGF	Sigma-Aldrich	Cat#E9644
Gastrin I (human)	Tocris	Cat#3006
Corning Matrigel Basement Membrane Matrix, LDEV-free	Corning	Cat#354234
5-Fluorouracil (5-FU)	Accord Healthcare	Cat#606544.3
Irinotecan	Accord Healthcare	Cat#713386.5
Dasatinib	Selleckchem	Cat#S1021
Verteporfin	Selleckchem	Cat#S1786
D-Luciferin	Goldbio	Cat#LUCK
PhosSTOP phosphatase inhibitor cocktail	Roche	Cat#PHOSS-RO

cOmplete Mini protease inhibitor cocktail	Roche	Cat#11836170001
DPX mountant	Sigma-Aldrich	Cat#06522
DAPI Fluoromount-G	Southern Biotech	Cat#0100-20
Protein A-Sepharose CL-4B	GE Healthcare	Cat#17-0780-01
Protein G-Sepharose 4 Fast Flow	GE Healthcare	Cat#17-0618-01
**Critical Commercial Assays**		
Dako Envision+ System-HRP Labelled Polymer anti-Rabbit	Agilent	Cat#K4003
Envision+ System-HRP Labelled Polymer anti-Mouse	Agilent	Cat#K4001
Dako Liquid DAB+ Substrate Chromogen System	Agilent	Cat#K3468
TSA Plus Cyanine 3/Fluorescein System	PerkinElmer	Cat#NEL753001KT
EZ-ECL Chemiluminescence Detection Kit for HRP	Biological Industries	Cat#20-500-120
ECL Prime Western Blotting System	GE Healthcare	Cat#RPN2232
CellTiter-Glo Luminescent Cell Viability Assay	Promega	Cat#G7571
APC BrdU Flow Kit	BD Biosciences	Cat#552598
Senescence β-Galactosidase Staining Kit	Cell Signaling	Cat#9860S
Cell Event Senescence Green Flow Cytometry Assay KiT	Invitrogen	Cat#C10840
CometAssay Kit	Trevigen	Cat#4250-050-K

RNeasy Micro Kit	Qiagen	Cat#74004
RT-First Strand cDNA Synthesis Kit	GE Healthcare Life Sciences	Cat#27-9261-01
SYBR Green I Master Kit	Roche	Cat#04887352001
Annexin V Apoptosis Detection Kit APC	Invitrogen	Cat#88-8007
Lenti-X Concentrator	Clontech	Cat#631232
**Experimental Models: Cell Lines**		
Human: HEK293T	ATCC	CRL-11268
Human: HCT116	ATCC	CCL-247
Human: Ls174T	ATCC	CL-188
Human: SW480	ATCC	CCL-228
Human: HT29	ATCC	HTB-38D
**Experimental Models: Organisms/Strains**		
Mouse: athymic nude mice (strain: Hsd:Athymic Nude-Foxn1nu)	Envigo	Cat#069
Mouse: NSG (strain: ANB//NOD.Cg-Prkdcscid Il2rgtm1Wjl/SzJ)	The Jackson Laboratory	JAX: 005557
**Oligonucleotides**		
Primers for RT-qPCR, see Table S7	This study	N/A
Primers for Chip-qPCR, see Table S7	This study	N/A
gRNA against *TP53*, see Table S7	This study	N/A
**Recombinant DNA**		
Plasmid: pMD2.G	From Trono Lab, unpublished	Addgene plasmid #12259
Plasmid: pCMV-dR8.2 dvpr	Stewart et al., 2003	Addgene plasmid #8455
Plasmid: lentiCRISPR v2	Sanjana et al., 2014	Addgene plasmid #52961

Plasmid: pLEX-hFLiG	Celià-Terrassa & Kang, 2018	N/A
Plasmid: pLTPC-H2BeGFP	Gift from Héctor G. Palmer Lab, unpublished	N/A
**Software and Algorithms**		
GraphPad Prism 6	Graphpad Software	https://www.graphpad.com; RRID:SCR_002798
Fiji (Image J)	Schneider et al., 2012	https://www.fiji.sc; RRID: SCR_002285
Benchling CRISPR design	Benchling	https://www.benchling.com; RRID: SCR_013955
FlowJo 10.6.2	BD Biosciences	https://www.flowjo.com; RRID: SCR_008520
Simplicity 4.2	Berthold	https://www.berthold.com/
LightCycler 480 Software 1.5.1.62	Roche	http://www.roche-applied-science.com/shop/products/absolute-quantification-with-the-lightcycler-carousel-based-system; RRID: SCR_012155
Olympus CellSens Standard 1.16	Olympus LifeSciences	https://www.olympus-lifescience.com/es/software/cellsens/
NIS-Elements AR 4.51.01	Nikon	https://www.microscope.healthcare.nikon.com/
BD FACSDiva Software 9.0	BD Biosciences	https://www.bdbiosciences.com/en-eu/products/software/instrument-software/bd-facsdiva-software
LAS X Life Science	Leica	https://www.leica-microsystems.com/
Adobe Photoshop and Illustrator CS4	Adobe Software	https://www.adobe.com/products/photoshop.html; RRID: SCR_014199
RStudio	RStudio Team	https://rstudio.com/
GSEA	Broad Institute	https://www.gsea-msigdb.org/gsea/index.jsp
ggplot	Bioconductor	
Corrplot	CRAN	https://cran.r-project.org/web/packages/corrplot/index.html
Survimer	CRAN	https://CRAN.R-project.org/package=survminer
Survival	CRAN	https://CRAN.R-project.org/package=survival
Heatmaply	CRAN	https://CRAN.R-project.org/package=heatmaply
Pheatmap	CRAN	https://CRAN.R-project.org/package=pheatmap

Limma	Bioconductor	https://bioconductor.org/packages/release/bioc/html/limma.html
DESeq2 R package	Bioconductor	https://bioconductor.org/packages/release/bioc/html/DESeq2.html
TopHat	Kim et al 2013	https://ccb.jhu.edu/software/tophat/index.shtml
HTSeq	Anders et al. 2015	https://htseq.readthedocs.io/en/master/

Supplementary Table S5. List of oligonucleotides for RT-qPCR and ChIP-qPCR and sgRNA for CIRSPR/Cas9 knockout used in this study.

Primers for RT-qPCR		
**Target**	**Forward**	**Reverse**
TP53	CTTTGAGGTGCGTGTTTGTG	GGGCAGTGCTCGCTTAGT
CDKN1A	CCGAAGTCAGTTCCTTGTGGA	TGGTGTCTCGGTGACAAAGT
MDM2	GCCATTGAACCTTGTGTGATT	GGCAGGGCTTATTCCTTTTC
PHLDA3	CAGCTGTGGAAGCGGAAG	GCGAAGCTGAGCTCCTTG
PLK2	AATAACAAAGTCTACGCCGCA	TCTTTGTCAATCTTTTCCCTTTG
ZMAT3	CTAGGGCAAAGCGCAAATAG	GACCAGCCACTCCAAAAGAG
SESN1	TGACCTGATGCCTTTCCTTC	CCTGGGGCTTAGTACCTTCC
LAPTM5	TCTTTTCCATCGCCTTCATC	CCTTCTGGAGCATCTTGGAG
TIMP2	TTCCCTCCCTCAAAGACTGA	CAAAGCCACCTACCTCCAAA
CRIP2	CGGTGGGCAGCTACATCTAT	CTGAGCACTCTCCCAGCAGA
KIFC3	TGCCATGTACGAGTCAGAGC	CGGTTCTTGTCCTCTTCCAG
MRAS	ACCGAGTTTTCCCATCAGTG	TCTCTTTCCCTCCCAGGTTT
SERPINH1	CTTCATGGTGACTCGGTCCT	CGATTTGCAGCTTTTCCTTC
CD99L2	CGGGTTGACATGAGAAAGGT	ATTCTGGCTTTGATGCTCGT
TUBB6	TGAGGGGCCACAAAATAAAC	TATAAGGCAACACGGCACAA
TPM2	GGACAGAGGATGAGGTGGAA	GCATCAGTGGCCTTCTTCTC
GLIPR1	CGCCATCACAAACTGGTATG	ATCTGCCCAAACAACCTGAG
TSPAN4	TGCCTCCTGCTCACTTTCTT	GTCTTGCTGGGCATACCTGT
ICAM1	GAAGTGGCCCTCCATAGACA	TCAAGGGTTGGGGTCAGTAG
ARL4C	TGAGTCCCTGCCTATTGTCC	CAGATGGGCTGCTAGGTTTC
VAMP5	CCTGAAGGAGAAGCCAAATG	GTCAAGGGAGAGCAAACACC
GPC1	CCCTACGCTCATCTCTGGAA	GACCTTGTGGAGGAAGGACA
COL18A1	GAGGGACAAGTGGACTCAGG	TTGGCTTCACATCACACACA
AGMAT	TCTTTCTGGGAACACAGCCC	CGGTTGTCACTTTGGGGAGA
KCNK5	GAGGTGTGAGTCTGCGGAAG	GCCCTCGATGTAGTTCCACC
CDX1	ACCTCCTCTCCAATGCCTGT	AGACTCGGACCAGACCTCCT
HPRT1	ATAAGCCAGACTTTGTTGG	ATAGGACTCCAGATGTTTCC
TBP	GGAAGTGACATTATCAACGC	CCAAGAAACAGTGATGCTG
ACTB	GCACCACACCTTCTACAATGAGC	TAGCACAGCCTGGATAGCAACG
**Primers for ChIP-qPCR**		
**Target**	**Forward**	**Reverse**
MDM2	GGGCAGGTTGACTCAGCTTTT	AGCTGGGAAAATGCATGGTTTA
BAX	GGTTCCTGGCTCTCTGATCC	AGGCTGGGCCTGTATCCTAC
CDKN1A	AGCAGGCTGTGGCTCTGATT	CAAAATAGCCACCAGCCTCTTCT
ZMAT3	CAAATTGCCACAAACATTCTGC	CTGGGGGAGACACATGCTAGA
**sgRNA for CRISPR/Cas9 knockout**		
**Target**	**sgRNA sequence**	
TP53 – Guide 1	CACCGTCGACGCTAGGATCTGACTG	
TP53 – Guide 2	CACCGACCAGCAGCTCCTACACCGG	
TP53 – Guide 3	CACCGCCATTGTTCAATATCGTCCG	
YAP1 – Guide 1	CACCGTCAGATCGTGCACGTCCGCG	
YAP1 – Guide 2	CACCGGAATGAGCTCGAACATGCTG	
YAP1 – Guide 3	CACCGTGTCGAAGATGCTGAGCTGT	

Supplementary Table S6. Tissue microarray (TMA) samples used in this study. Samples obtained at diagnosis. The corresponding clinical data and H-score of nuclear YAP1 is indicated.

Patient nº	Tumour Location	Clinical TNM	Treatment	nYAP1 Hscore	OS (mo)	DFS (mo)
**1**	Sigma	T3N0	Surgery	8,3	42,6	42,6
**2**	Rectum-Sigma	T3N2M1 lung	Chemotherapy + Targeted therapy	135,0	47,8	47,8
**3**	Sigma	T4N2	Chemotherapy	126,7	77,0	77,0
**4**	Right colon	T3N1	Chemotherapy	160,0	27,3	27,3
**5**	Splenic angle	T4N2	Surgery	240,0	6,0	6,0
**6**	Right colon	T2NO	Chemotherapy	40,0	75,0	75,0
**7**	Sigma	T3N1	Surgery	70,0	58,9	58,9
**8**	Sigma	T3N1	Chemotherapy	20,0	69,0	69,0
**9**	Sigma	T3N1	Chemotherapy	63,3	74,0	74,0
**10**	Sigma	N/A	Surgery	46,7	67,9	67,9
**11**	Right colon	T4N1	Surgery	0,0	20,3	15,2
**12**	Sigma	T3N2	Surgery	190,0	56,9	23,3
**13**	Sigma	T3N0	Surgery	43,3	61,8	61,8
**14**	Rectum	T2N2	Chemotherapy+Radiotherapy	66,7	70,0	32,5
**15**	Sigma	T3NX	Chemotherapy	60,0	12,2	35,6
**16**	Sigma	T3N0	Surgery	3,3	7,1	7,1
**17**	Sigma	T3N0	Surgery	0,0	74,0	74,0
**18**	Rectum	T3N0	Surgery	40,0	23,4	8,2
**19**	Sigma	T3N0	Surgery	33,3	36,5	36,5
**20**	Right colon	T3N0	Surgery	6,7	51,8	51,8
**21**	Sigma	T3N0	Surgery	3,3	67,0	67,0
**22**	Sigma	T3N0	Surgery	0,0	74,1	74,1
**23**	Ascendent colon	T3N2	Chemotherapy	30,0	30,4	30,4
**24**	Sigma	T3N0	Surgery	73,3	72,0	72,0
**25**	Right colon	T3N2	Chemotherapy	93,3	67,0	67,0
**26**	Right colon	T3N0	Surgery	3,3	60,9	32,5
**27**	Sigma	T3N2	Chemotherapy+Targeted therapy	93,3	24,3	10,1
**28**	Right colon	T3N0	Surgery	53,3	63,9	63,9
**29**	Right colon	T4N0	Chemotherapy	53,3	64,9	64,9
**30**	Sigma	T4N0	Chemotherapy	5,0	26,4	26,4
**31**	Right colon	T4N0	Surgery	0,0	2,0	2,0
**32**	Sigma	T3N0	Surgery	6,7	26,4	26,4
**33**	Descendent colon	T4N0	N/A	25,0	29,4	25,3
**34**	Rectum-Sigma	T3N0	Surgery	46,7	67,9	67,9
**35**	Rectum-Sigma	T3N0	Surgery	130,0	67,9	67,9
**36**	Sigma	T3N1	Chemotherapy+Radiotherapy	0,0	46,7	5,1
**37**	Right colon	T4N1	Surgery	100,0	22,3	13,2
**38**	Sigma	T4N1	Surgery	40,0	28,4	28,4
**39**	Sigma	T3N1	Chemotherapy	N/A	62,9	62,9
**40**	Rectum	T3N0	Chemotherapy+Radiotherapy	146,7	64,9	64,9
**41**	Rectum-Sigma	T2N0	Chemotherapy+Radiotherapy	96,7	67,9	67,9

**42**	Right colon	T2N1	Chemotherapy	12,5	70,0	70,0
**43**	Sigma	T4N1	Chemotherapy	0,0	63,9	63,9
**44**	Splenic angle	T4N1	Surgery	103,3	21,3	9,1
**45**	Right colon	T3N0	N/A	120,0	33,5	33,5
**46**	Right colon	T3N1	Surgery	20,0	5,0	5,0
**47**	Right colon	T4N2	Surgery	0,0	5,1	3,0
**48**	Right colon	T3N0	Surgery	5,0	65,0	65,0
**49**	Sigma	T4N0	Chemotherapy	0,0	64,9	64,9
**50**	Right colon	T3N2	Chemotherapy	3,3	15,2	11,2
**51**	Sigma	T4N1	Chemotherapy	22,5	66,9	66,9
**52**	Sigma	T4N1	Chemotherapy+Targeted therapy	12,5	29,4	14,2
**53**	Descendent colon	T2N1	Surgery	20,0	65,9	65,9
**54**	Sigma	T3N2	Surgery	10,0	10,1	6,0
**55**	Sigma	T4N2	Surgery	48,3	60,9	18,2
**56**	Right colon	T3N0	Surgery	45,0	71,0	71,0
**57**	Sigma	T3N0	Surgery	2,5	75,1	75,1
**58**	Sigma	T3N1	Surgery	0,0	55,8	55,8
**59**	Hepatic angle	T3N0	Surgery	0,0	66,0	66,0
**60**	Rectum-Sigma	T3N0	Surgery	N/A	28,4	28,4
**61**	Right colon	T3N0	Surgery	6,7	62,9	62,9
**62**	Right colon	T3N2	Chemotherapy	73,3	68,0	68,0
**63**	Right colon	T3N0	Surgery	65,0	1,0	1,0
**64**	Right colon	T3N1	Chemotherapy	27,5	52,8	52,8
**65**	Sigma	T3N0	Surgery	0,0	74,0	22,4
**66**	Right colon	T3N1	Chemotherapy	45,0	72,0	18,3
**67**	Right colon	T3N0	Surgery	1,7	72,0	72,0
**68**	Rectum-Sigma	T4N2	Chemotherapy+Radiotherapy	160,0	16,3	15,2
**69**	Rectum	T3N0	Surgery	66,7	70,0	70,0
**70**	Sigma	T3N2	Chemotherapy	60,0	70,0	70,0
**71**	Right colon	T3N0	Surgery	75,0	65,9	65,9
**72**	Splenic angle	T3N0	Surgery	160,0	10,1	10,1
**73**	Sigma	T3N0	Surgery	3,3	39,5	39,5
**74**	Rectum	T4N0	Chemotherapy+Radiotherapy	37,5	63,9	63,9
**75**	Right colon	T3N1	Chemotherapy+Targeted therapy	43,3	70,0	70,0
**76**	Sigma	T3N1	Chemotherapy	63,3	75,0	75,0
**77**	Rectum	T4N1	No treatment	0,0	14,2	11,1
**78**	Right colon	T3N0	Surgery	23,3	41,6	13,2
**79**	Right colon	T4N2	Surgery	N/A	37,5	33,5
**80**	Descendent colon	T3N2	Chemotherapy	3,3	68,0	68,0
**81**	Sigma	T4N0	Chemotherapy	18,3	60,9	60,9
**82**	Sigma	T4N1	Chemotherapy	3,3	65,9	37,6
**83**	Sigma	T4N0	Chemotherapy	33,3	62,9	62,9
**84**	Right colon	T3N0	Surgery	0,0	67,0	67,0
**85**	Right colon	T4N0	Surgery	20,0	30,4	8,1
**86**	Descendent colon	T4N1	Chemotherapy	16,7	63,9	63,9
**87**	Rectum	T3N1	Chemotherapy+Radiotherapy	166,7	67,9	39,6
**88**	Sigma	T3N1	Chemotherapy	26,7	59,8	59,8
**89**	Right colon	T3N0	Surgery	3,3	0,0	0,0

**90**	Descendent colon	T3N1	Chemotherapy	26,7	50,7	50,7
**91**	Descendent colon	T3N1	Chemotherapy	3,3	71,0	71,0
**92**	Sigma	T3N0	Surgery	3,3	65,9	65,9
**93**	Right colon	T3N0	Surgery	36,7	72,1	72,1
**94**	Right colon	T3N0	Surgery	6,7	25,4	25,4
**95**	Right colon	T3N0	Chemotherapy+Radiotherapy	23,3	52,8	32,5
**96**	Right colon	T3N0	Chemotherapy	3,3	61,9	61,9
**97**	Right colon	T3N0	Surgery	100,0	64,9	64,9
**98**	Sigma	T3N0	Surgery	1,0	10,1	10,1
**99**	Sigma	T3N0	Surgery	2,5	60,9	60,9
**100**	Right colon	T3N1	Chemotherapy	0,0	61,9	61,9
**101**	Rectum-Sigma	T3N0	Surgery	2,5	62,9	62,9
**102**	Sigma	T3N0	Surgery	13,3	60,9	60,9
**103**	Sigma	T3N2	Chemotherapy+Targeted therapy	100,0	63,9	3,0
**104**	Sigma	T3N1	Chemotherapy	106,7	24,3	24,3
**105**	Sigma	T4N1	Chemotherapy	58,3	64,9	64,9
**106**	Right colon	T3N1	Chemotherapy	86,7	3,0	3,0
**107**	Right colon	T3N1	Chemotherapy	40,0	61,9	61,9
**108**	Right colon	T3N0	N/A	6,7	33,5	33,5
**109**	Transverse colon	T3N0	Surgery	3,3	67,0	67,0
**110**	Right colon	T3N0	Surgery	63,3	32,5	13,2
**111**	Right colon	T3N0	Surgery	66,7	59,8	59,8
**112**	Splenic angle	T3N0	Surgery	5,0	64,9	64,9
**113**	Sigma	T3N0	Surgery	80,0	50,7	8,2
**114**	Sigma	T3N0	Surgery	63,3	52,8	52,8
**115**	Hepatic angle	T3N0	Surgery	30,0	58,8	58,8
**116**	Right colon	T3N0	Surgery	45,0	54,8	54,8
**117**	Rectum-Sigma	T3N0	Chemotherapy+Radiotherapy	140,0	60,9	60,9
**118**	Rectum	T2N1	Chemotherapy+Radiotherapy	40,0	63,9	63,9
**119**	Sigma	T3N0	Surgery	40,0	53,8	53,8
**120**	Descendent colon	T3N0	Surgery	140,0	54,8	54,8
**121**	Right colon	T4N2	Chemotherapy	83,3	18,2	10,1
**122**	Splenic angle	T3N1	Surgery	46,7	61,8	61,8
**123**	Rectum-Sigma	T3N0	Chemotherapy	103,3	60,9	60,9
**124**	Sigma	T3N1	Chemotherapy	10,0	59,8	59,8
**125**	Sigma	T3N2	Chemotherapy+Targeted therapy	41,7	42,6	14,1
**126**	Rectum-Sigma	T4N0	Surgery	20,0	31,5	23,3
**127**	Right colon	T3N0	Surgery	18,3	58,8	58,8
**128**	Right colon	T4N0	Surgery	10,7	55,8	55,8
**129**	Descendent colon	T3N0	Surgery	0,0	56,8	9,1
**130**	Rectum	T3N0	Chemotherapy+Radiotherapy	16,7	56,8	56,8
**131**	Descendent colon	T3N0	Surgery	23,3	51,7	51,7
**132**	Rectum-Sigma	T2N0	Chemotherapy+Radiotherapy	0,0	48,7	48,7
**133**	Right colon	T3N0	Surgery	33,3	67,0	67,0
**134**	Right colon	T3N0	Surgery	50,0	49,7	49,7
**135**	Splenic angle	T4N0	Surgery	4,0	58,8	58,8
**136**	Rectum	T2N0	Chemotherapy+Radiotherapy	N/A	19,3	19,3
**137**	Rectum-Sigma	T1N0	Chemotherapy+Radiotherapy	110,0	54,8	54,8

**138**	Rectum-Sigma	T2N0	Chemotherapy+Radiotherapy	15,0	54,8	54,8
**139**	Right colon	T3N1	Chemotherapy	50,0	58,8	58,8
**140**	Sigma	T4N2	Surgery	160,0	0,0	0,0
**141**	Ascendent colon	T3N0	Surgery	56,7	58,8	58,8
**142**	Sigma	T3N0	Surgery	15,0	35,5	35,5
**143**	Rectum-Sigma	T3N1	Chemotherapy+Radiotherapy	50,0	28,5	11,2
**144**	Right colon	T3N2	Chemotherapy	20,0	52,8	52,8
**145**	Right colon	T4N2	Chemotherapy	8,3	53,8	53,8
**146**	Descendent colon	T4N1	Chemotherapy	35,0	58,8	30,5
**147**	Right colon	T4N1	Surgery	0,0	54,8	54,8
**148**	Rectum-Sigma	T3N1	Chemotherapy	93,3	58,8	58,8
**149**	Sigma	T1N1	Chemotherapy	1,7	57,8	57,8
**150**	Sigma	T3N2	Chemotherapy	8,3	56,8	56,8
**151**	Right colon	T4N1	Surgery	0,0	52,8	52,8
**152**	Right colon	T4N1	Chemotherapy	160,0	54,7	54,7
**153**	Descendent colon	T3N1	Chemotherapy	1,7	56,8	15,2
**154**	Sigma	T3N2	Surgery	100,0	66,0	16,2
**155**	Sigma	T3N1	Chemotherapy	90,0	57,8	57,8
**156**	Sigma	T1N0	Chemotherapy	5,0	20,3	20,3
**157**	Caecum	T3N2	Chemotherapy	21,7	56,8	56,8
**158**	Right colon	T2N1	Chemotherapy	50,0	63,8	63,8
**159**	Right colon	T3N0	Surgery	0,0	52,7	52,7
**160**	Right colon	T3N0	Surgery	17,0	13,2	7,1
**161**	Sigma	T3N0	Surgery	60,0	51,8	51,8
**162**	Sigma	T3N0	Surgery	12,5	59,8	59,8
**163**	Descendent colon	T3N0	Surgery	6,7	57,8	57,8
**164**	Rectum-Sigma	T2N0	Radiotherapy	110,0	55,8	55,8
**165**	Descendent colon	T3N2	Chemotherapy+Radiotherapy	63,3	41,6	41,6
**166**	Rectum-Sigma	T3N2	Chemotherapy	6,7	48,7	48,7
**167**	Sigma	T4N2	Chemotherapy	153,3	58,8	58,8
**168**	Sigma	T4N0	Chemotherapy	26,7	60,9	60,9
**169**	Right colon	T2N1	Chemotherapy	8,3	55,8	55,8
**170**	Right colon	T3N1	Chemotherapy	0,0	52,7	52,7
**171**	Sigma	T3N1	Surgery	11,7	56,9	56,9
**172**	Ascendent colon	T4N1	Chemotherapy	36,7	58,8	58,8
**173**	Right colon	T3N0	Surgery	0,0	55,8	55,8
**174**	Transverse colon	T4N1	Chemotherapy	106,7	59,8	59,8
**175**	Right colon	T3N1	Chemotherapy	6,7	61,9	38,6
**176**	Sigma	T4N2	Chemotherapy	N/A	62,9	33,5
**177**	Sigma	T3N0	Surgery	50,0	56,8	56,8
**178**	Right colon	T4N0	Chemotherapy	60,0	57,8	57,8
**179**	Splenic angle	T3N0	Surgery	1,7	61,9	61,9
**180**	Descendent colon	T3N0	Chemotherapy	0,0	62,8	62,8
**181**	Sigma	T3N2	Surgery	86,7	1,0	1,0
**182**	Rectum-Sigma	T3N2	Chemotherapy	63,3	49,6	49,6
**183**	Sigma	T4N0	Chemotherapy	56,7	60,9	60,9
**184**	Ascendent colon	T3N0	Surgery	4,0	52,7	52,7
**185**	Rectum-Sigma	T3N1	Chemotherapy	0,0	64,9	25,3

**186**	Right colon	T3N0	Surgery	N/A	25,4	14,2
**187**	Descendent colon	T3N0	Chemotherapy	66,7	68,0	68,0
**188**	Sigma	T4N0	Chemotherapy	110,0	51,7	13,2
**189**	Right colon	T4N0	Surgery	0,0	54,8	54,8
**190**	Sigma	T2N1	Chemotherapy	5,0	52,7	52,7
**191**	Right colon	T3N0	Surgery	26,7	53,8	53,8
**192**	Rectum-Sigma	T3N1	Surgery	0,0	1,0	1,0
**193**	Right colon	T3N0	Surgery	21,7	0,0	0,0
**194**	Right colon	T3N0	Surgery	33,3	61,9	61,9
