# Supplementary material for: p53 wild-type colorectal cancer cells that express a fetal gene signature are associated with metastasis and poor prognosis
Source: Nat Commun. 2022 May 23;13:2866. doi: 10.1038/s41467-022-30382-9 (PMC9126967; doi:10.1038/s41467-022-30382-9)
Supplement: Supplementary file 3 — Description of Additional Supplementary Information [file 41467_2022_30382_MOESM3_ESM.docx]

**Inventory of Supporting Information**

The supplementary information zip file include:

1. ***The supplementary information pdf*** which contains:

**Supplementary Figures:**

- Supplementary Figure 1: Low-dose CT treatment induces a quiescent-like state to CRC PDO in the absence of persistent DNA damage and senescence.
- Supplementary Figure 2: PQL cells retain tumor initiating capacity
- Supplementary Figure 3: Low-dose CT induces a robust p53 signaling
- Supplementary Figure 4. . Acquisition of quiescent phenotype by CT treatment in patient samples.
- Supplementary Figure 5: Acquisition of a restricted feISC signature by CT and p53 dependency.
- Supplementary Figure 6: Identification of a fetal ISC signature with prognostic value in cancer.

**Supplementary Tables:**

- Supplementary table 1: Patient-derived organoids and CRC cells lines used in this study
- Supplementary table 2: Human gastrointestinal tumor samples used in this study
- Supplementary table 3: Cox proportional hazards analysis of the feISC signature
- Supplementary table 4: Materials table
- Supplementary table 5: List of oligonucleotides for RT-qPCR and ChIP-qPCR and sgRNA for CIRSPR/Cas9 knockout used in this study
- Supplementary table 6: Tissue microarray (TMA) samples used in this study

1. ***The supplementary data file 1*** is an excel spreadsheet containing the differentially expressed genes between IC20 or IC30 and control PDO5; IC30 and control PDO66; and treated-PDO5/PDO66 analyzed together removing the bath effect.
2. ***The supplementary data file 2*** is an excel spreadsheet containing the expression correlation matrix from CT induced feISC genes in the Marisa (Marisa et al., 2013) dataset.
3. ***The uncropped scans blots figures 1j-k, 4a.***
4. ***The reporting summary***
