# Supplementary material for: p53 wild-type colorectal cancer cells that express a fetal gene signature are associated with metastasis and poor prognosis
Source: Nat Commun. 2022 May 23;13:2866. doi: 10.1038/s41467-022-30382-9 (PMC9126967; doi:10.1038/s41467-022-30382-9)

Uncropped scans of all blots in Supplementary Information

Figure S1j: anti-γH2AX

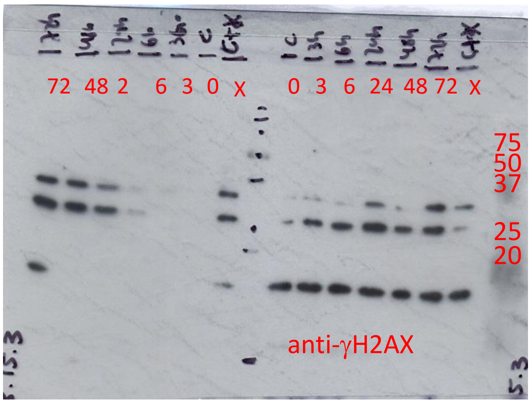

Figure S1j: anti-H3

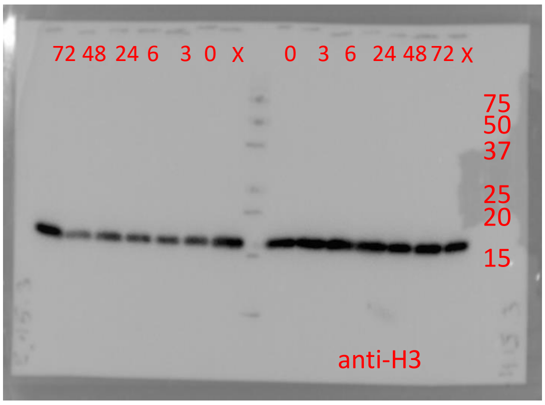

Figure S1k: anti-γH2AX

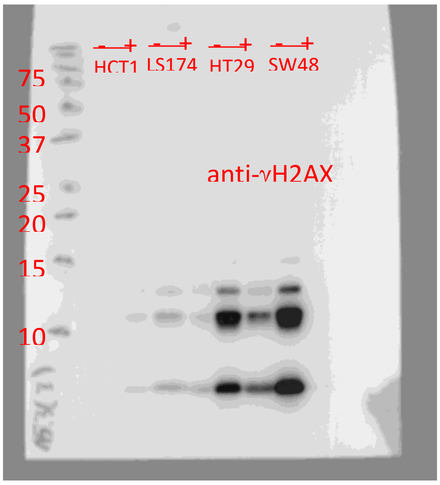

Figure S1k: anti-H3

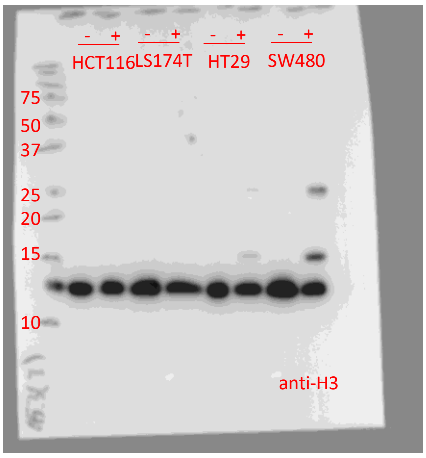

Figure S4a: anti-YAP1

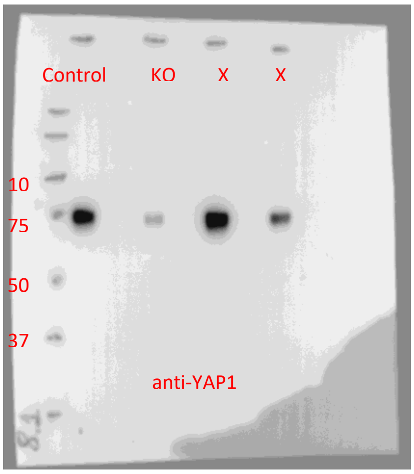

Figure S4a: anti-Tubulin

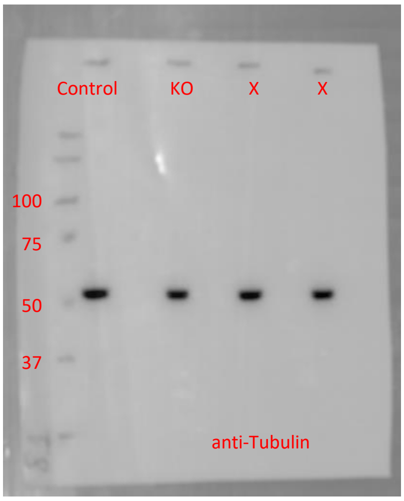

Supplement: Supplementary file 6 — Uncropped scans of all blots [file 41467_2022_30382_MOESM6_ESM.pdf]
