# Supplementary material for: p53 wild-type colorectal cancer cells that express a fetal gene signature are associated with metastasis and poor prognosis
Source: Nat Commun. 2022 May 23;13:2866. doi: 10.1038/s41467-022-30382-9 (PMC9126967; doi:10.1038/s41467-022-30382-9)
Supplement: Supplementary file 7 — Reporting Summary [file 41467_2022_30382_MOESM7_ESM.pdf]

nature portfolio

Corresponding author(s): NCOMMS-21-12440C

Last updated by author(s): 2022/04/12

Reporting Summary

Nature Portfolio wishes to improve the reproducibility of the work that we publish. This form provides structure for consistency and transparency in reporting. For further information on Nature Portfolio policies, see our Editorial Policies and the Editorial Policy Checklist.

Statistics

For all statistical analyses, confirm that the following items are present in the figure legend, table legend, main text, or Methods section.

n/a	Confirmed	
☐	☒	The exact sample size (*n*) for each experimental group/condition, given as a discrete number and unit of measurement
☐	☒	A statement on whether measurements were taken from distinct samples or whether the same sample was measured repeatedly
☐	☒	The statistical test(s) used AND whether they are one- or two-sided<br>*Only common tests should be described solely by name; describe more complex techniques in the Methods section.*
☒	☐	A description of all covariates tested
☒	☐	A description of any assumptions or corrections, such as tests of normality and adjustment for multiple comparisons
☐	☒	A full description of the statistical parameters including central tendency (e.g. means) or other basic estimates (e.g. regression coefficient) AND variation (e.g. standard deviation) or associated estimates of uncertainty (e.g. confidence intervals)
☐	☒	For null hypothesis testing, the test statistic (e.g. *F*, *t*, *r*) with confidence intervals, effect sizes, degrees of freedom and *P* value noted<br>*Give P values as exact values whenever suitable.*
☒	☐	For Bayesian analysis, information on the choice of priors and Markov chain Monte Carlo settings
☒	☐	For hierarchical and complex designs, identification of the appropriate level for tests and full reporting of outcomes
☐	☒	Estimates of effect sizes (e.g. Cohen's *d*, Pearson's *r*), indicating how they were calculated

Our web collection on statistics for biologists contains articles on many of the points above.

Software and code

Policy information about availability of computer code

Data collection	-NIS-Elements AR (v.4.51.01)<br>-Leica Application Suite v(2.7.3.9723)<br>-Olympus CellSens Standard (v.1.16)<br>-BD FACS Diva Software (v.9.0)<br>-Cytovision software (v.7.0)<br>-Simplicity (v.4.2)<br>-Illumina HiSeq2000<br>-CANCERTOOL (website platform)
Data analysis	-fastQC (v.0.11.8)<br>-Trimmomatic (v.0.39)<br>-TopHat (v.2.1.1)<br>-HTSeq (v.0.9.1)<br>-Samtools (v.1.9)<br>-DESeq2 R package (v.1.30.1) (Love et al, 2014)<br>-removeBatchEffect from the limma package (v.3.46.0)<br>-ChIPseeker package (v.1.26.0)<br>-Integrative Genomics Viewer (IGV) (v.2.8.13)<br>-R (v.XXXX) (heatmaply and pheatmap (Galili et al, 2018), survival (v.3.2-3), survimer (v.0.4.8), corrplot package (v.0.84), ggplot packages)<br>-GSEA (v.4.1.0)<br>-GraphPad Prism 6<br>-Fiji (opencomet plugin)<br>-Benchling online platform<br>-FlowJo (v.10.6.2)

-LightCycler 480 (v.1.5.1.62)
-Adobe PhotoShop and Illustrator CS4

For manuscripts utilizing custom algorithms or software that are central to the research but not yet described in published literature, software must be made available to editors and reviewers. We strongly encourage code deposition in a community repository (e.g. GitHub). See the Nature Portfolio guidelines for submitting code & software for further information.

Data

Policy information about availability of data

All manuscripts must include a data availability statement. This statement should provide the following information, where applicable:
- Accession codes, unique identifiers, or web links for publicly available datasets
- A description of any restrictions on data availability
- For clinical datasets or third party data, please ensure that the statement adheres to our policy

-All data associated with this study are present in the paper or the Supplementary Materials.
-No new codes were used in this study.
-RNA sequencing and ChIP sequencing data have been deposited in NCBI's Gene Expression and are accessible through GEO Series accession no. GSE155354 [https://www.ncbi.nlm.nih.gov/geo/query/acc.cgi?acc=GSE155354] and no. GSE164161 [https://www.ncbi.nlm.nih.gov/geo/query/acc.cgi?acc=GSE164161] respectively.
-Public data sets used in this study are also accessible through GEO Series accession no. GSE39582 [https://www.ncbi.nlm.nih.gov/geo/query/acc.cgi?acc=GSE39582] for Marisa data set and GSE14333 [https://www.ncbi.nlm.nih.gov/geo/query/acc.cgi?acc=GSE14333] for Jorissen data set; and for TCGA data set through the TCGA portal [http://www.tcgaportal.org/]. In this study, data from these data sets have been obtained using CANCERTOOL platform [http://genomics.cicbiogune.es/CANCERTOOL/].

Field-specific reporting

Please select the one below that is the best fit for your research. If you are not sure, read the appropriate sections before making your selection.

☒ Life sciences ☐ Behavioural & social sciences ☐ Ecological, evolutionary & environmental sciences

For a reference copy of the document with all sections, see nature.com/documents/nr-reporting-summary-flat.pdf

Life sciences study design

All studies must disclose on these points even when the disclosure is negative.

Sample size	We did not use sample size calculation to predetermine sample size. Instead, sample sizes were chosen based on established and accepted standards of the field. In general, in vitro analyses were repeated two to three times to ensure reproducible conclusions.
Data exclusions	No data exclusion
Replication	All experiments were replicated as indicated in the respective figure legends.
Randomization	In all experiments animals were randomly ascribed to the different experimental groupss. For all in vitro studies, randomization is not relevant because all the cells were under identical conditions before different treatment samples applied.
Blinding	Investigators were blinded for animal group allocation. For cell-based experiments, cell conditions were known when preparing the samples and when analyzing the results.

Reporting for specific materials, systems and methods

We require information from authors about some types of materials, experimental systems and methods used in many studies. Here, indicate whether each material, system or method listed is relevant to your study. If you are not sure if a list item applies to your research, read the appropriate section before selecting a response.

Materials & experimental systems

n/a	Involved in the study	
☐	☒	Antibodies
☐	☒	Eukaryotic cell lines
☒	☐	Palaeontology and archaeology
☐	☒	Animals and other organisms
☐	☒	Human research participants
☒	☐	Clinical data
☒	☐	Dual use research of concern

Methods

n/a	Involved in the study	
☐	☒	ChIP-seq
☒	☐	Flow cytometry
☒	☐	MRI-based neuroimaging

Antibodies

Antibodies used	Mouse monoclonal anti-γH2AX (pS139) (clone N1-431 (RUO)) BD Biosciences Cat#564719; RRID:AB_2738913 – (dilution 1/1000)<br>Mouse monoclonal anti-Ki67 (clone MM1) Leica Biosystems Cat#NCL-Ki67-MM1; RRID:AB_442101 – (dilution 1/1000)<br>Rabbit polyclonal anti-Cleaved Caspase-3 (Asp175) Cell Signaling Cat#9661; RRID:AB_2341188 – (dilution 1/1000)<br>Mouse monoclonal anti-p53 (clone DO-1) Abcam Cat#ab1101; RRID:AB_297667 – (dilution 1/1000)<br>Rabbit monoclonal anti-p21 (clone EPR362) Abcam Cat#ab109520; RRID:AB_10860537 – (dilution 1/1000)<br>Rabbit monoclonal anti-CKN2A/p16INK4a (clone EPR1473) Abcam Cat#ab108349; RRID:AB_10858268 – (dilution 1/1000)<br>Goat polyclonal anti-EphB2 RD Systems Cat#AF467; RRID:AB_355375 – (dilution 1/1000)<br>Rabbit polyclonal anti-CD99L2 Abcam Cat#ab224164 – (dilution 1/1000)<br>Mouse monoclonal anti-TIMP2 (clone 3A4) Abcam Cat#ab1828; RRID:AB_2256129 – (dilution 1/1000)<br>Rabbit polyclonal anti-MRas Abcam Cat#ab26303; RRID:AB_470849 – (dilution 1/1000)<br>Rabbit polyclonal anti-TUBB6 Abcam Cat#PA5-P8948 – (dilution 1/2000)<br>Rabbit monoclonal anti-ICAM1 (clone EPR4776) Abcam Cat#ab109361; RRID:AB_10958467 – (dilution 1/1000)<br>Rabbit monoclonal anti-Hsp47 (clone EPR4217) (SERPINH1) Abcam Cat#ab109117; RRID:AB_10888995 – (dilution 1/1000)<br>Rabbit monoclonal anti-YAP1 (clone EP1674Y) Abcam Cat#ab52771; RRID:AB_2219141 – (dilution 1/1000)<br>Rabbit polyclonal anti-TSPAN4 Thermo Fisher Scientific Cat#PA5-69344; RRID:AB_2688603 – (dilution 1/1000)<br>Mouse monoclonal anti-S100A4 (clone CL0240) Atlas Antibodies Cat#AMAB90599; RRID:AB_2665603 – (dilution 1/1000)<br>Rabbit polyclonal anti-Histone H3 antibody-Nuclear Marker and ChIP Grade Abcam Cat#ab1791; RRID:AB_302613 – (dilution 1/10000)<br>Mouse monoclonal anti-alpha-Tubulin (clone B-5-1-2) Sigma-Aldrich Cat#T6074; RRID:AB_477582 – (dilution 1/10000)<br>Goat Anti-Rabbit Immunoglobulins/HRP antibody (2ary) Agilent Cat#P0448; RRID:AB_2617138 – (dilution 1/2000)<br>Rabbit Anti-Mouse Immunoglobulins/HRP antibody (2ary) Agilent Cat#P0260; RRID:AB_2636929 – (dilution 1/2000)<br>Polyclonal Rabbit Anti-Goat Immunoglobulins/HRP antibody (2ary) Agilent Cat#P0449; RRID:AB_2617143 – (dilution 1/2000)
Validation	anti-γH2AX: validated by company for Western Blotting and IHC and in several other publications from our laboratory.<br>anti-Ki67: validated by company for IHC human B cells.<br>anti-Cleaved Caspase-3: validated by company for Western blotting of extracts from human cells by appearance of a band at the corresponding size and in IHC of paraffin-embedded human and murine samples.<br>anti-p53: validated by company for Western Blotting (human) by appearance of a band at the corresponding size, for ChIP-seq in (HEK-293 cell chromatin) and by knockout validation; and ¬in this manuscript using knockout controls.<br>anti-p21: validated by company for Western Blotting (human) by appearance of a band at the corresponding size and by knockout validation.<br>anti-CKN2A/p16INK4a: validated by company for Western Blotting using human HeLa, HEK293T and Saos-2 cell lysates. Also validated using recombinant protein.<br>anti-EphB2: validated by company for IHC in MBA MB 468 Human Cell Line and in Embryonic Mouse Brain. Additionally validated in Washburn et al., 2020.<br>anti-CD99L2: validated by company for IHC in human cells.<br>anti-TIMP2: validated by company for IHC in human cells.<br>anti-MRas: validated by company for Western blotting of extracts from human cells by appearance of a band at the corresponding size.<br>anti-TUBB6: validated by company for Western blotting of extracts from human cells by appearance of a band at the corresponding size.<br>anti-ICAM1: validated by company for Western blotting of extracts from human cells by appearance of a band at the corresponding size and for IHC of human samples. Additionally validated in Li Q et al., 2021 and Yao MY et al., 2020.<br>anti-Hsp47 (SERPINH1): validated by company for Western blotting of extracts from HeLa cells by appearance of a band at the corresponding size, for IHC of human cells, and by knockout validation.<br>anti-YAP1: validated by company for Western blotting of extracts from HeLa cells by appearance of a band at the corresponding size and for IHC of human cells.<br>anti-TSPAN4: validated by company for Western blotting of extracts from human cells by appearance of a band at the corresponding size.<br>anti-S100A4: validated by company for Western blotting of extracts from human cells by appearance of a band at the corresponding size and for IHC of human samples.<br>anti-Histone H3: validated by company for Western blotting of extracts from human cells by appearance of a band at the corresponding size. Additionally validated by several other publications from our laboratory.<br>anti-alpha-Tubulin: validated by company for Western blotting of extracts from human cells by appearance of a band at the corresponding size. Additionally validated by several other publications from our laboratory.

Eukaryotic cell lines

Policy information about cell lines

Cell line source(s)	Human: HEK293T ATCC CRL-11268<br>Human: HCT116 ATCC CCL-247<br>Human: Ls174T ATCC CL-188<br>Human: SW480 ATCC CCL-228<br>Human: HT29 ATCC HTB-38D
Authentication	Cell lines were not authenticated
Mycoplasma contamination	All cell lines tested negative for mycoplasm contamination

Commonly misidentified lines (See ICLAC register)	none

Animals and other organisms

Policy information about studies involving animals; ARRIVE guidelines recommended for reporting animal research

Laboratory animals	NSG mice (strain: ANB//NOD.Cg-Prkdcscid Il2rgtm1Wjl/SzJ) (ten-week-old males) and athymic nude mice (strain: Hsd:Athymic Nude-Foxn1nu) mice (5-to-7-week-old males)
Wild animals	none
Field-collected samples	none
Ethics oversight	Procedures involving living animals were conducted under pathogen-free conditions and according to guidelines from the Animal Care Committee at the Generalitat de Catalunya. The Committee for Animal Experimentation at the Institute of Biomedical Research of Bellvitge (Barcelona) approved these studies.

Note that full information on the approval of the study protocol must also be provided in the manuscript.

Human research participants

Policy information about studies involving human research participants

Population characteristics	All patient data is listed in Supplementary Table S2 and S6
Recruitment	Patients were recruited based on clinical diagnosis of CRC without additional bias
Ethics oversight	Ethical Committee at Hospitao del Mar

Note that full information on the approval of the study protocol must also be provided in the manuscript.

ChIP-seq

Data deposition

☒ Confirm that both raw and final processed data have been deposited in a public database such as GEO.

☒ Confirm that you have deposited or provided access to graph files (e.g. BED files) for the called peaks.

Data access links *May remain private before publication.*	ChIP sequencing data have been deposited in NCBI's Gene Expression and are accessible through GEO Series accession no. GSE164161.
Files in database submission	Raw *fasq.gz files for:<br>p53 ChIP-seq from treated IC20 patient-derived organoid replicate 1<br>p53 ChIP-seq from treated IC20 patient-derived organoid replicate 2<br>Input from treated IC20 patient-derived organoid<br><br>Processed *narrowPeak files for:<br>p53 ChIP-seq from treated IC20 patient-derived organoid replicate 1<br>p53 ChIP-seq from treated IC20 patient-derived organoid replicate 2
Genome browser session (e.g. UCSC)	No longer applicable

Methodology

Replicates	ChIP-seq was performed in duplicated.
Sequencing depth	Total filtered sequences, which ranged between 32 and 55 million per sample, were aligned against the reference genome with Bowtie2
Antibodies	Mouse monoclonal anti-p53 DO-1 Abcam Cat#ab1101; RRID:AB_297667
Peak calling parameters	Peak annotation was performed with ChIPseeker and Annotatr packages; and functional enrichment analysis with enrichR, using the latest version of GO annotations. Peaks heatmaps and multiple coverage correlation across bigwig files were performed with Deeptools
Data quality	Peaks that were detected in the input samples were filtered out, as well as the black regions downloaded from the ENCODE portal
Software	MACS2 software was run for each replicate considering unique alignments (q-value < 0.1). Peaks from biological replicates were merged using bedtools
